# Effects of Zinc Ions Released From Ti-NW-Zn Surface on Osteogenesis and Angiogenesis *In Vitro* and in an *In Vivo* Zebrafish Model

**DOI:** 10.3389/fbioe.2022.848769

**Published:** 2022-04-21

**Authors:** Wen-Qing Zhu, Kang Li, Shan Su, Wei Chen, Yao Liu, Jing Qiu

**Affiliations:** ^1^ Department of Oral Implantology, Affiliated Stomatological Hospital of Nanjing Medical University, Nanjing, China; ^2^ Jiangsu Province Key Laboratory of Oral Diseases, Nanjing, China; ^3^ Jiangsu Province Engineering Research Center of Stomatological Translational Medicine, Nanjing, China

**Keywords:** Ti-NW-Zn surfaces, zebrafish, angiogenesis, osteogenesis, MAPK/ERK signaling pathway

## Abstract

Zinc-modified titanium materials have been widely applied in oral implants. Among them, our previous studies have also successfully prepared a novel acid-etched microstructured titanium surface modified with zinc-containing nanowires (Ti-NW-Zn) and proved its excellent biocompatibility. It is well known that the functional regulation between angiogenesis and osteogenesis is of great importance for bone remodeling around implants. However, there are few reports concerning the biological safety of zinc ions released from materials and the appropriate concentration of released zinc ions which was more conducive to angiogenesis and bone regeneration. In this study, we investigated the effects of zinc ions released from Ti-NW-Zn surfaces on angiogenesis and osteogenesis using the zebrafish model and revealed the relationship between angiogenesis and osteogenesis via HUVECs and MC3T3-E1s *in vitro*. We found that the zinc ions released from Ti-NW-Zn surfaces, with a concentration lower than median lethal concentrations (LCs) of zebrafish, were biologically safe and promote osteogenesis and angiogenesis *in vivo*. Moreover, the proper concentration of zinc ions could induce the proliferation of HUVECs and osteogenic differentiation. The positive effects of the appropriate concentration of zinc ions on osteoblast behaviors might be regulated by activating the MAPK/ERK signaling pathway. These aspects may provide new sights into the mechanisms underlying zinc-modified titanium surfaces between osteogenesis and angiogenesis, to lay the foundation for further improving the materials, meanwhile, promoting the applications in dentistry.

## Introduction

Titanium (Ti) and its alloys as bio-implants have excellent biocompatibility and osteogenic properties after chemical composition and topography modification using various methods (Gong et al., 2017; Ma et al., 2018; Zhou et al., 2020). Among them, zinc-coated titanium materials have been extensively used in biomedicine and have improved osteoblastic differentiation (Huo et al., 2013; Jin et al., 2014). Our previous studies reported the preparation of a novel acid-etched microstructured titanium surface modified with zinc-containing nanowires (Ti-NW-Zn) (Zhu et al., 2019a). We showed this Ti-NW-Zn surface has excellent biocompatibility and an osteogenic capacity and exhibits excellent corrosion resistance under a complex oral electrochemical environment. However, the internal mechanism of this surface on osteogenesis requires additional clarification with particular emphasis on the effect of zinc introduced onto the Ti-NW-Zn surface on bone development.

Zinc is an essential trace element, required for the functional integrity of many organ systems and for development, growth, and tissue repair (Ryu et al., 2009; Shen et al., 2014). Zinc ions induce and stimulate the expression of genes related to osteoblastic differentiation and bone formation and stimulate angiogenesis *in vitro* and *in vivo* (Yu et al., 2017; Song et al., 2020). Studies have shown that bone regeneration is inseparable from angiogenesis (Gao et al., 2020). New blood vessels in the bone transport oxygen, nutrients, and metabolites; they also serve as important pathways in the transport of related cell signaling molecules and maintain bone regeneration and repair as a whole microenvironment (Kanczler and Oreffo, 2008; Shi et al., 2014). Studies on zinc-modified titanium surfaces have focused on promoting bone formation, while surface exploration to regulate the vascularization direction has not been reported. During implant osseointegration, osteogenesis and angiogenesis are complementary and indispensable (Chen et al., 2018). Therefore, in-depth research on the relationship between osteogenesis and angiogenesis upon exposure to Zn ions is crucial, especially the role of angiogenesis for *in vivo* and *in vitro* bone regeneration.

*In vivo* experimental models with a particular emphasis on rabbits and dogs have been used to simulate oral implantations and investigate the characteristics and the basic molecular mechanisms of osseointegration (Guillot et al., 2016; Li et al., 2018; Yu et al., 2019). However, the complexity of metabolic and physiochemical regulations of higher vertebrates makes it difficult to dissect the early phase of vascularized bone regeneration within a short period upon exposure to zinc ions. A simpler animal model in lower vertebrates may help extend knowledge in this field*.*

Zebrafish have emerged as an alternative *in vivo* model to study angiogenesis, a tissue-specific germ line-induced transgenic line that promotes enhanced green fluorescent protein (EGFP) expression in all endothelial cells under a friend leukemia integration-1 (Fli-1) promoter. The Tg (Fli-1:EGFP)^y1^ transgenic zebrafish expresses EGFP in endothelial cells during early embryonic vascular development, making it possible to capture images of vascular development and adult blood vessels in real time (Lawson and Weinstein, 2002; Wu et al., 2020). Recently, adult zebrafish have gained importance as innovative and readily available resources for studying skeletal systems and bone metabolisms at the cellular and molecular levels (Pasqualetti et al., 2012; Carnovali et al., 2019). In particular, the caudal fins represent a unique anatomical feature because they form from a type of dermal bone and include osteoclasts, osteoblasts, and other characteristics of bone tissue, which approximates the human lamellar bone (Chang et al., 2018; Sehring and Weidinger, 2020). Caudal fins have a high regenerative capacity (Li et al., 2021; Banu et al., 2022). These characteristics make zebrafish an ideal model to visualize angiogenesis and easily observe mineral matrix deposition and resorption (Ny et al., 2006). The same approach does not apply to internal bones in other higher vertebrate animals.

In this study, we aim to investigate the positive effects of zinc ions released from Ti-NW-Zn surfaces on angiogenesis *via* the Tg (Fli-1:EGFP)^y1^ zebrafish embryo assay and on the osteogenesis via adult zebrafish caudal fins model of bone regeneration. Based on *in vivo* results, we used endthelial cells and osteoblasts as *in vitro* models to explore the cellular and genetic regulatory mechanisms of the angiogenesis effects on osteogenesis under zinc ion exposure. This study will assess the appropriate zinc ion concentration released from Ti-NW-Zn surfaces for the promotion of osteogenesis and angiogenesis and should provide a scientific basis for the functional optimization design of zinc-modified titanium surfaces and their potential transformation applications.

## Materials and Methods

### Material Preparation and Characterization

The preparation of Ti-NW-Zn surfaces and the identification of surface elements were described previously (Zhu et al., 2019a). The samples were randomly assigned to 6-well plates with 2 ml of DI H_2_O after washing and drying.

The surface morphologies of cp-Ti, Ti-NW, and Ti-NW-Zn samples were observed by field emission scanning electron microscopy (SEM, Ultra 55, Zeiss, Germany). X-ray photoelectron spectroscopy (XPS, Thermo Scientific Escalab 250Xi, United States) was used to identify surface constituents and bonding energies. The hydrophilicity of samples was evaluated from contact angles by testing a droplet of sessile distilled water on the substrates by an Automatic Contact Angle Meter Model (SL200B, Kino, United States). All measurements were performed in triplicate.

### Zinc Ion Release Assay

In accordance with some reports and our previous studies (Ming et al., 2017; Polo-Montalvo et al., 2021; Tang et al., 2021), Ti-NW-Zn samples were immersed in 6-well plates (2 ml PBS/well) at 37°C for 1 h and 1, 4, and 7 d. The concentrations of zinc ions in the PBS solutions were quantified using a Zinc Assay Kit (E011-1-1; Nanjing Jiancheng Bioengineering Institute, Nanjing, China). The absorbance was measured using a microplate reader (SpectraMax 190, MD, United States) at 630 nm. Three samples of each group were used in this assay.

### Zebrafish Embryo Collection

Wild-type zebrafish (*Danio rerio*) embryos were obtained from wild-type AB strain adult zebrafish, while Tg (Fli-1:EGFP)^y1^ zebrafish embryos were obtained from outcrosses of Tg (Fli-1:EGFP)^y1^ parents (Wu et al., 2020). The spawning adults were offspring of parents obtained from the Model Animal Research Center of Nanjing University and maintained in an aquatic animal breeding and reproduction system (HAISHENG, Shanghai, China) under standard conditions. All zebrafish studies were approved by the Institutional Animal Care and Use Committee at Nanjing Medical University. Groups consisting of one male and two females were mated in translucent plastic tanks, and embryos were obtained within 30 min after the onset of light in the morning. The eggs were collected immediately after fertilization, washed, and collected by E3 medium in 6-well plates at 28.5°C.

### Zebrafish Rearing

Wild-type AB strain adult zebrafish (*Danio rerio*) were maintained in a recirculating aquatic system at 28.5°C with a 10/14-h dark/light cycle according to previously reported standards (Wu et al., 2020). The zebrafish were reared in an aquatic animal breeding and reproduction system (HAISHENG, Shanghai, China) under standard conditions. Male and female zebrafish were randomly used in these experiments.

### Angiogenesis Observation of Transgenic Zebrafish Embryo

After 24 hpf (hours-post-fertilization), Tg (Fli‐1:EGFP)^y1^ zebrafish embryos were pretreated with 75 nM vascular endothelial growth factor receptor-2 tyrosine kinase inhibitor (VRI) to inhibit normal angiogenesis for 8 h and then co-cultured on Ti-NW-Zn surfaces randomly preassigned to 6-well plates (10 embryos per well containing 2 ml medium) at 28.5°C for 3 d to detect defective vessel regenerations. The zebrafish were anesthetized (4% Tricaine) after 48 hpf, and the intersegmental blood vessels (ISVs) were observed under the inverted fluorescent microscope. Gross morphological changes in the sub-intestinal vessels (SIVs) of the yolk sac region were observed under the inverted fluorescent microscope at 72 hpf. Three parameters indicative of angiogenesis or vasodilation were measured: variation in the number of vessels, vessel thickness, and the subintestinal venous plexus (SIVP) branching (angiogenic phenotype).

### Survival Test in Embryos and Adult Fish

Based on the release of zinc ions from Ti-NW-Zn surfaces, all embryos were cultured in 6-well plates (10 embryos per well containing 2 ml medium) at 28.5°C with different concentrations of zinc ions, including a nominal concentration of 0 (control group), as well as 2, 4, 8, 16, 24, and 32 ppm zinc ions, which last for 120 hpf (N = 10 for each testing concentration). LC50 tests were conducted. Adult fish were exposed to DI H_2_O with different zinc ion concentrations, including a control group containing no zinc ions, and concentrations of 1 and 2 ppm zinc ions for 7 d for chronic exposures (N = 5 for each testing concentration). LC50 tests were also conducted according to these series groups. The exposure water was changed daily. The zebrafish were fasted throughout these experiments.

### Zebrafish Fin Amputation and Regeneration Test

Adult zebrafish between 7 and 10 months with body weights of 0.3–0.5 g were initially anesthetized with Tricaine (160 mg/L) for 5 min, and then caudal fins were partially amputated using a #11 blade. All fish were recovered in an open tank for 2 h and randomly assigned to 500-ml culture vessels with different zinc ion concentrations, where one was placed in each vessel and the solution was changed every 3 d and fed twice a day. Then, Alizarin Red stain was used to detect the skeletal calcification of zebrafish fins. The experiment continued for 9 d and repeated three times.

### Cell Culture

A commercially available osteoblast-like cell line MC3T3-E1 (Cell Bank of Chinese Academy of Science, Shanghai, China) and human umbilical vein endothelial cells (HUVECs, ATCC, United States) were used in this study. MC3T3-E1 cells were cultured in *α*-minimum essential medium (*α*-MEM; Gibco, United States) supplemented with 10% fetal bovine serum (FBS; Gibco, United States) and 1% penicillin/streptomycin (Gibco, United States). HUVECs were cultured in Dulbecco’s modified Eagle’s medium (DMEM; Gibco, United States), which contained 10% fetal bovine serum (Gibco, United States) and 1% penicillin/streptomycin (Gibco, United States). Both MC3T3-E1 and HUVECs were maintained in an incubator containing 5% CO_2_ and 95% air at 37°C. The fresh complete medium was changed every 2 days. Upon reaching 80% cell confluence, the cells were passaged every three or 4 days.

### Collection and Preparation of Conditioned Medium

HUVECs were seeded in the 6-well plates and incubated with Zn ions at doses of 0, 1, 2, 4, and 8 ppm. When the cell confluence reached 80%, the culture medium of each group was collected and centrifuged (1,000 rpm, 15 min) under sterile conditions. After collecting the supernatant, it was mixed with *α*-MEM containing 10% FBS and 1% penicillin/streptomycin in a 1:1 ratio to obtain a conditioned medium (CM), which was placed in a –20°C refrigerator for later use.

### Cell Proliferation Assay

A CCK-8 kit was used to assess cell proliferation. MC3T3-E1 cells (3×10^3^ cells/well) and HUVECs (3×10^3^ cells/well) were seeded in the 96-well plates and treated with Zn ions at different concentrations (0, 1, 2, 4, and 8 ppm). MC3T3-E1 cells were cultured for 1, 3, and 6 days, while HUVECs were cultured for 1, 2, and 3 days. Afterward, 100 μl fresh medium containing 10 μl of CCK-8 (Beyotime, Shanghai, China) was added to each well and incubated for 2 h at 37°C. The absorbance of each well was measured by using a microplate spectrophotometer (Spectramax 190, CA, United States) at 450 nm. The assay was carried out in triplicate.

### Cell Adhesion and Spreading Assay

Commercially pure titanium (99.5 wt.%, Alfa Aesar, United States) disks were polished with 600-, 800-, 1,200-, and 1500-grit silicon carbide abrasive papers. MC3T3-E1 cells (5×10^3^ cells/well) were seeded on the surface of polished titanium disks in the 96-well plates and treated with or without CM as described above for 8 h. Afterward, each sample was washed with PBS and fixed with 4% paraformaldehyde at room temperature for 10 min. To observe cell morphology on titanium, the cells were stained with 100 nM rhodamine phalloidin (Cytoskeleton, United States) for 30 min and 4′,6′-diamidino-2-phenylindole (DAPI; Beyotime, Shanghai, China) for 2 min in the dark. Nine fields were randomly selected from each sample to observe cell morphology under a laser scanning confocal fluorescence microscope (LSM710NLO; Zeiss, Jena, Germany) at ×100 and ×200 magnifications. Then, we counted the cell numbers in each field and calculated the average value. Quantification analysis defined as “cell numbers” was performed by ImageJ and GraphPad Prism 9.1.1 software and repeated three times.

### Immunofluorescence Staining for FAK and VCAM-1

MC3T3-E1 cells were seeded on 24-well glass coverslips (1×10^4^ cells/well) and cultured with different concentrations of zinc ions. After 8 h, the samples were washed with ice-cold PBS, fixed with 4% paraformaldehyde for 30 min at room temperature, and incubated with 0.5% Triton X-100 (Beyotime, Shanghai, China) for 15 min. Then, the samples were incubated with 10% goat serum for 1 h at 37°C to block the non-specific antibody-binding sites. The cells were incubated with rabbit monoclonal antibodies against FAK and VCAM-1 (1:100 in PBS; Cell Signaling Technology, Beverly, MA, United States) overnight and fluorescein isothiocyanate-conjugated goat anti-rabbit IgG (1:200 in PBS) at 37°C for 1 h. The nucleus was stained with DAPI (C1002; Beyotime, China) for 3 min as follows. Finally, the signal was visualized and captured by fluorescence microscopy (DM4000M; Leica, Stuttgart, Germany). The assay was performed in triplicate.

### Western Blotting

MC3T3-E1 cells (2×10^5^ cells/well) were seeded in 6-well plates and cultured with CM as described before, after which the cells were washed with precooled PBS and lysed with RIPA buffer containing 1% PMSF. Protein samples were separated by electrophoresis, transferred to PVDF membranes (Millipore, Billerica, MA, United States), blocked in protein-free rapid blocking buffer (EpiZyme, Shanghai, China) for 10 min, and incubated with primary antibodies specific for Runx2 (1:1,000; 12556, CST, United States), OSX (1:1,000; ab22552; Abcam, United States), OCN (1:1,000; ab93876, Abcam, United States), ERK (1:1,000; 4,695, CST, United States), p-ERK (1:1,000; 4,370, CST, United States), and GAPDH (1:1,000; BM0627, Boster, China) at 4°C overnight. Afterward, the PVDF membranes were incubated with secondary antibodies (1:3,000; ZB-2301; Goat anti-Rabbit IgG, ZSGB-BIO, China) for 2 h at room temperature and exposed to the ECL substrate (NCM Biotech, Suzhou, China). GAPDH served as a loading control. All experiments were performed in triplicate. Quantification was performed by ImageJ and GraphPad Prism 9.1.1 software and repeated three times.

### Statistics

Statistical analyses were performed by SPSS 22.0 software (SPSS, Inc., Chicago, IL, United States) using one-way analysis of variance (ANOVA) with the Student–Newman–Keuls (SNK) method for multiple comparisons. The significant changes were set as *p < 0.05, **p < 0.01.

## Results

### Surface Characterization

**FIGURE 1:**
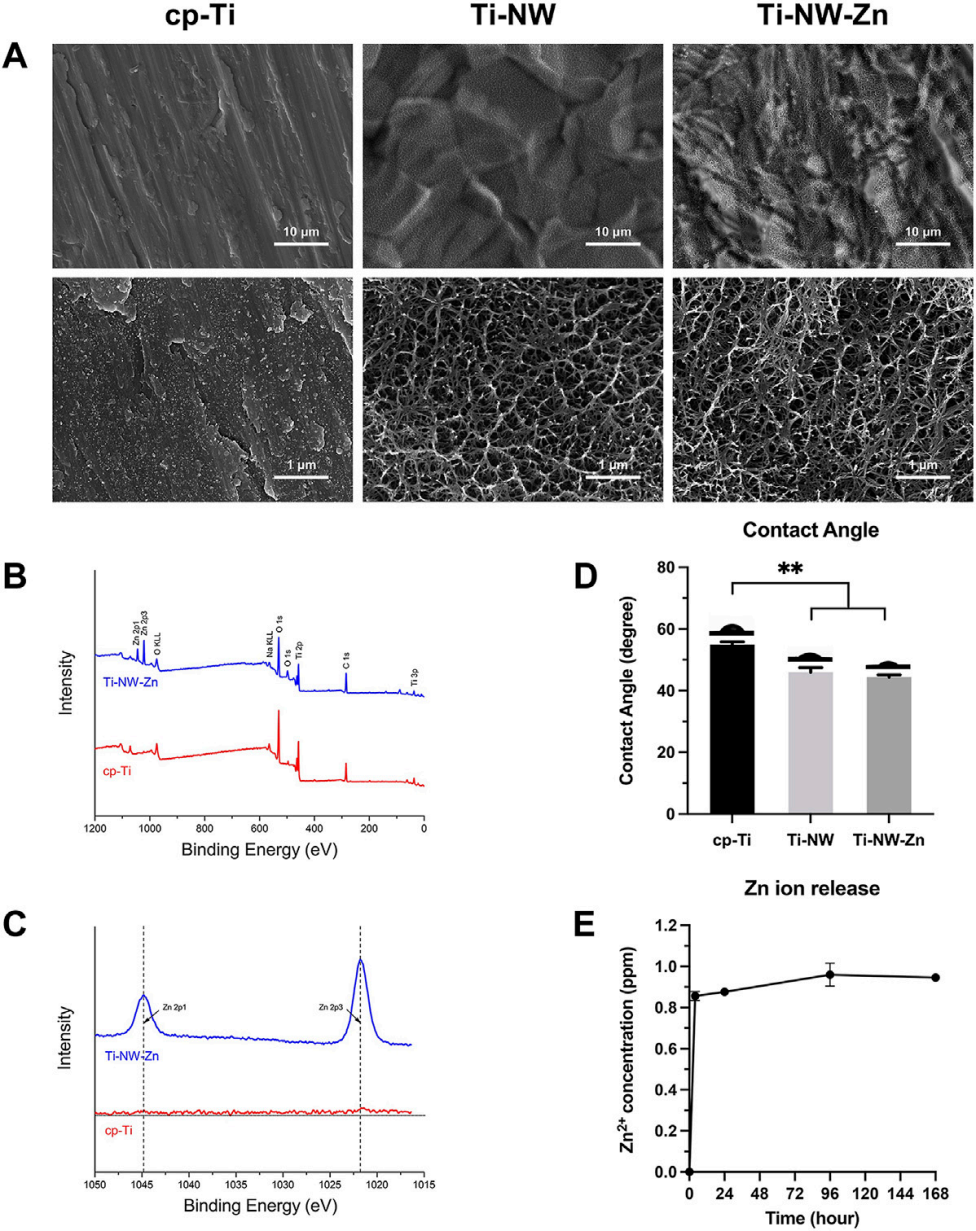
Surface characterization of different titanium surfaces. **(A)** SEM images of surface morphologies of cp-Ti, Ti-NW, and Ti-NW-Zn. Upper panel: ×5000 magnification. Lower panel: ×50000 magnification; **(B)** XPS wide scan spectra analysis of cp-Ti and Ti-NW-Zn surfaces; **(C)** XPS high-resolution spectra analysis of Zn 2p; **(D)** contact angles of cp-Ti, Ti-NW, and Ti-NW-Zn surfaces. Results were presented as mean ± SD (*p < 0.05, **p < 0.01; N = 3); **(E)** concentrations of Zn ion released from Ti-NW-Zn samples into PBS after 4 h and 1, 4, and 7 days; N = 3.

SEM images of the sample surfaces are shown in Figure 1A. The cp-Ti surface was relatively smooth, and scratches produced in the polishing step can be seen at lower magnification, while the morphology of Ti-NW and Ti-NW-Zn surfaces appeared similar, which presented uniformly rough, with groove-like structures on it. At higher magnification, the porous nanostructures were observed on the Ti-NW and Ti-NW-Zn surfaces, interweaved into networks.

The results of X-ray photoelectron spectroscopy showed the elemental composition of the samples. The wide scan spectrum (Figure 1B) showed that all samples were mainly composed of titanium, oxygen, and carbon. Zinc peaks were detected on the Ti-NW-Zn surface but not on the cp-Ti surface, indicating that zinc was incorporated into the modified titanium surface. The results of high-resolution spectra were shown in Figure 1C, which presented two peaks at 1,044.8 eV (Zn 2p1) and 1,021.8 eV (Zn 2p3), verifying the existence of divalent zinc on the modified titanium surface.

Meanwhile, the contact angles of the cp-Ti, Ti-NW, and Ti-NW-Zn surfaces were about 55°, 45°, and 44°, respectively (Figure 1D). And there were no significant differences in the contact angles between Ti-NW and Ti-NW-Zn surfaces. The modified titanium surfaces were more hydrophilic than the cp-Ti surfaces.

In order to clarify the promoting effects of the Ti-NW-Zn surface on osteogenesis and angiogenesis, we further detect the amount of zinc ions released from the Ti-NW-Zn surface. Figure 1E shows the concentrations of zinc ions released from Ti-NW-Zn surfaces into PBS. It was found that the samples released 0.9 ppm of zinc ions within 24 h, respectively. After the burst release in the initial 24-h period, concentrations of zinc ions released from samples nearly reached peaks. Then, the Zn ions were gradually released into PBS in the ensuing time.

### Promoting Effect of Ti-NW-Zn Surface on Angiogenesis

**FIGURE 2:**
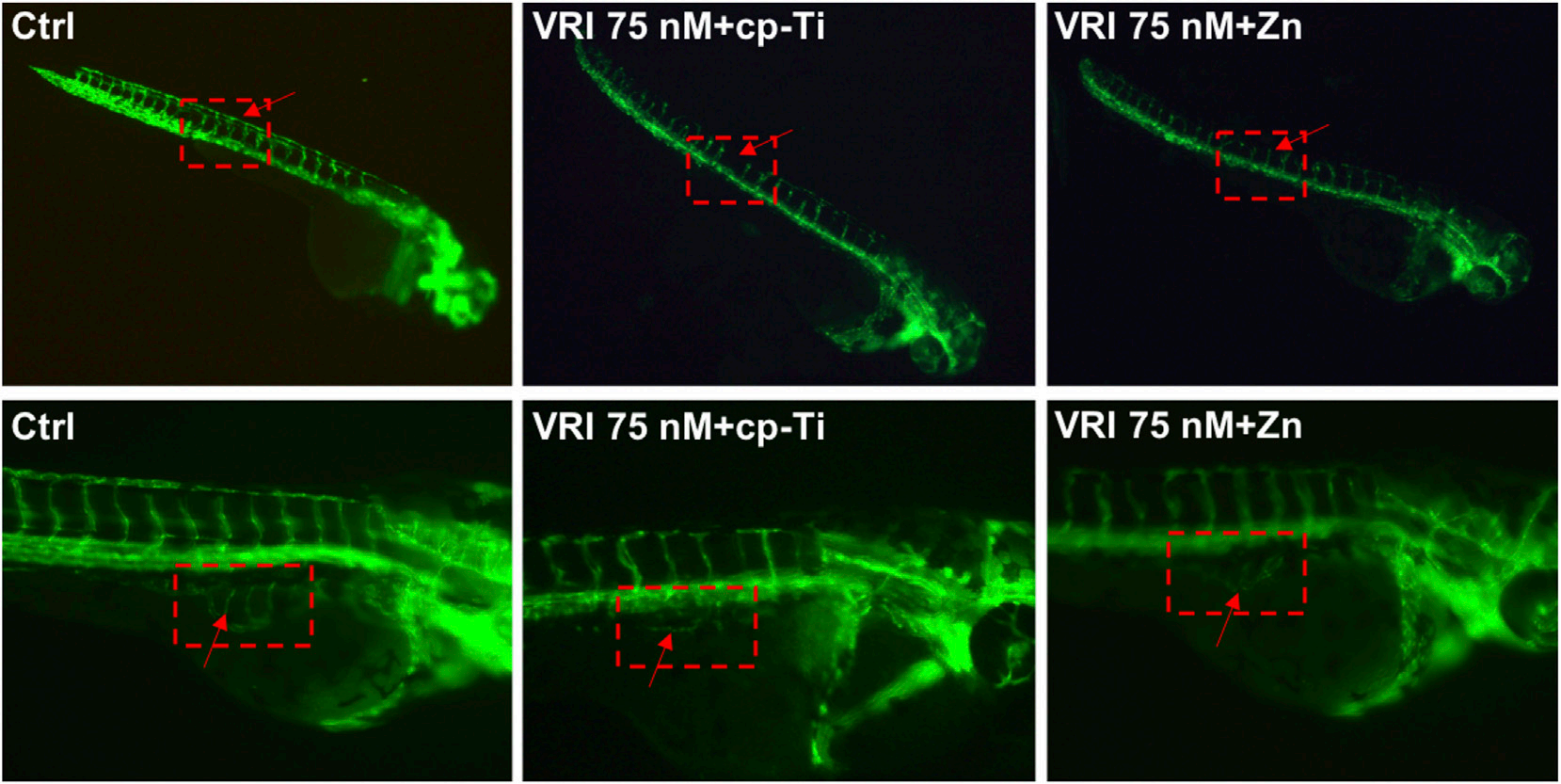
Angiogenesis of transgenic zebrafish in the group of control, cp-Ti, and Ti-NW-Zn.

After pretreatment with 75 nM vascular endothelial growth factor receptor-2 tyrosine kinase inhibitor (VRI) to inhibit the normal embryo angiogenesis for 8 h and incubation on the cp-Ti (commercially pure titanium) surface or Ti-NW-Zn surface for 24 h, the intersegmental blood vessels (ISVs) of Tg (Fli-1:EGFP)^y1^ zebrafish were observed via an inverted fluorescent microscope. As shown in Figure 2, the number of ISVs in the medial region of Tg (Fli-1:EGFP)^y1^ zebrafish on the cp-Ti surface decreased compared to the control group, and the ISVs were thinner and showed little morphological distortion, but Tg (Fli-1:EGFP)^y1^ zebrafish on the Ti-NW-Zn surface showed more ISVs than the cp-Ti surface group while less than the control group. Gross morphological changes in the sub-intestinal vessels (SIVs) of the yolk sac region were also observed in the 72 hpf, and we found that SIVs of zebrafish on the cp-Ti surface are nearly absent after being pretreated with VRI, while SIVs of zebrafish on the Ti-NW-Zn surface showed some new vessel branching; these results indicated that the Ti-NW-Zn surface can promote the zebrafish angiogenesis.

### Promoting Effect of Ti-NW-Zn Surface on Osteogenesis

**FIGURE 3:**
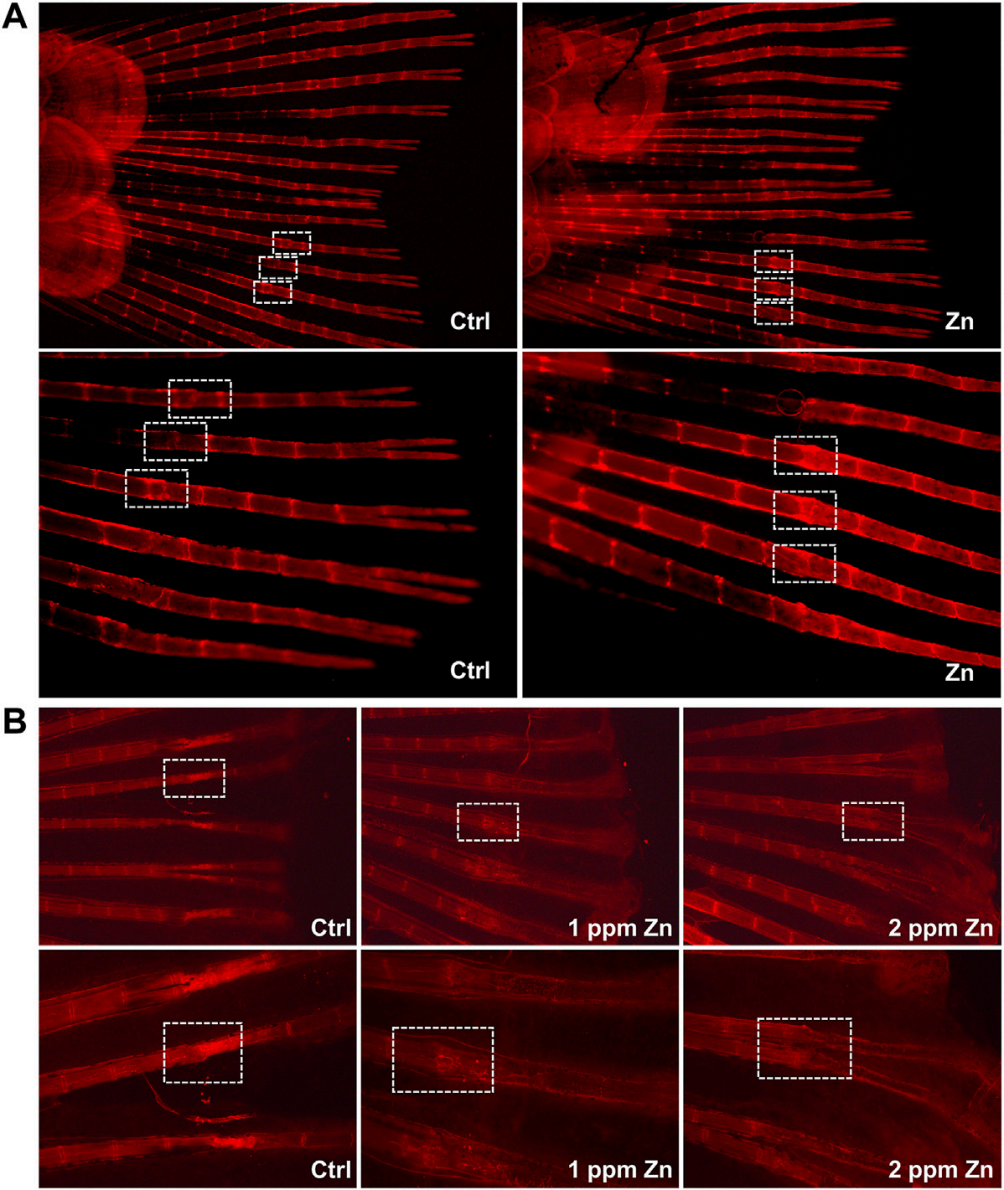
Zebrafish fin amputation and regeneration test. **(A)** Effect of the control group and Ti-NW-Zn surface on osteogenesis; **(B)** effect of different concentrations of zinc ions on osteogenesis: 0, 1, and 2 ppm.

Since blood vessels and bone development are closely connected, and we have found that the Ti-NW-Zn surface can promote the zebrafish angiogenesis, we further attempted to explore the effect of the Ti-NW-Zn surface on osteogenesis *in vivo* and constructed the zebrafish caudal fin regeneration model. Then, 9 days after caudal fin amputation, the regeneration of the caudal fins was visible to the naked eye. Afterward, Alizarin Red stain was used to detect the skeletal mineralization of zebrafish. As shown in Figure 3A, we can clearly find the fracture healing lines in the fin rays. Interestingly, in the control group, the width of the new fin rays at the fracture was significantly reduced compared to the original parts, while zebrafish on the Ti-NW-Zn surface showed obvious bulges in the fractured area. Moreover, the staining of the fracture on the Ti-NW-Zn surface was also deeper than the control group, suggesting that the mineralization was more adequate on the Ti-NW-Zn surface. These results indicated that the Ti-NW-Zn surface can promote zebrafish bone regeneration and mineralization.

### Effects of Different Zinc Ion Concentrations on Osteogenesis

To find out the most suitable zinc ion concentration to promote osteogenesis *in vivo*, we further studied the effects of different concentrations of zinc ions on the regeneration and development of zebrafish caudal fins. In Figure 3B, compared with the control group, 1 ppm zinc ions significantly promoted bone bulges in the fractured area, which showed a better trend of bone healing. However, the 2 ppm zinc ions seemed to inhibit the fracture healing of the fin rays, where the width of the new fin rays at the fracture was significantly reduced, the structure at the fracture was abnormal, and the bone mineral density at the fracture was reduced. These results revealed that different concentrations of zinc ions showed different effects on bone development, and 1 ppm zinc ions in this study were more conducive to bone development.

### Mortality Curve and Median Lethal Concentration for Zinc Ion Exposure

**FIGURE 4:**
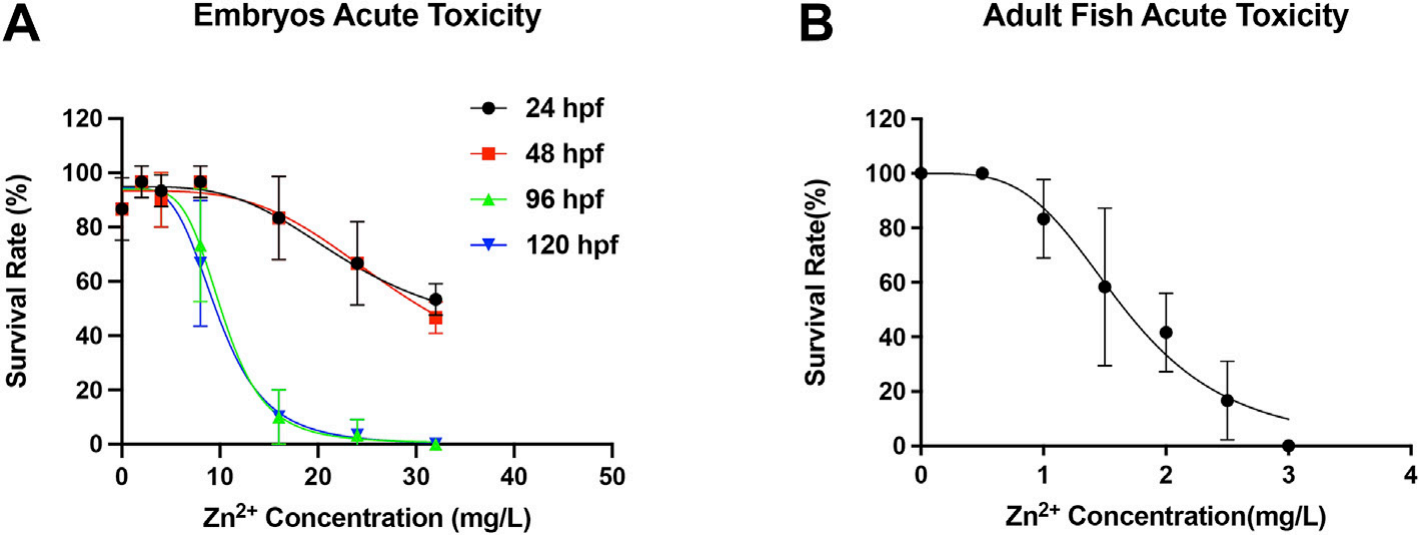
Survival rate in **(A)** embryos (N = 10) and **(B)** adult fish (N = 5).

The survival test of embryos was performed at different concentrations (0, 2, 4, 8, 16, 24, and 32 ppm) of zinc ions, from low to high concentrations for 120 h of exposure (Figure 4A). No deaths occurred in the control group throughout the exposure experiments. Embryos incubated at zinc ion concentrations of 1 ppm or lower showed mortality similar to the control group, while embryos treated with 32 ppm had 100% mortality after 24 h of incubation. Median lethal concentration (LC) was calculated from the percentage of embryo mortality for each zinc ion concentration with different exposure times (Figure 4A). The 120-h LC50 test of zinc ions was estimated at around 10 ppm. Similarly, we also explored the survival test of adult fish exposure to different concentrations of zinc ions (0, 0.5, 1, 1.5, 2, 2.5, and 3 ppm). As shown in Figure 4B, adult fish exposed to zinc ion concentrations of 1 ppm or lower showed no deaths, while adult fish treated with 3 ppm had 100% mortality after 24 h of incubation. These results indicated the effect of zinc ion concentration was dose-dependent and time-dependent, which had different effects on individuals at different developmental stages. Interestingly, the reason why embryos showed higher resistance to zinc ion exposure needed further exploration.

### Cell Proliferation

**FIGURE 5:**
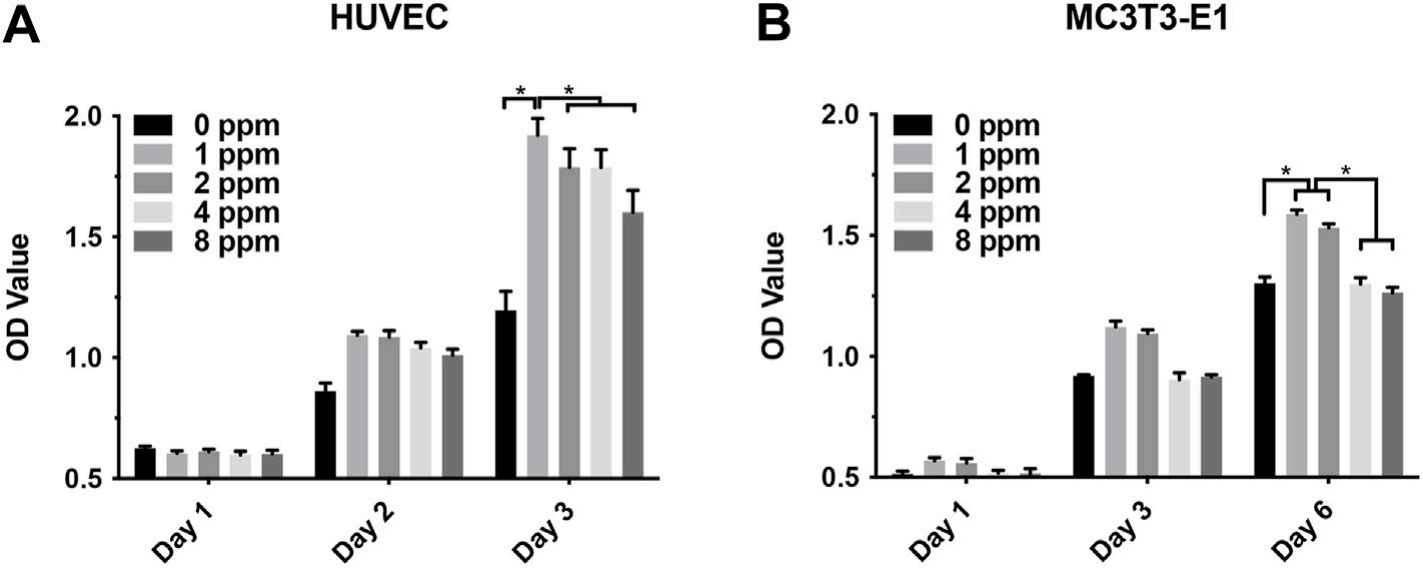
**(A)** Proliferation of human umbilical vein endothelial cells HUVEC with different concentrations of zinc ions for 1, 2, and 3 days; **(B)** proliferation of osteoblast-like MC3T3-E1 cells for 1, 3, and 6 days. Results were presented as mean ± SD (*p < 0.05,**p < 0.01; N = 3).

The results of cell proliferation assessed using the CCK-8 assay is shown in Figure 5. On the first day, no obvious proliferation changes in HUVECs and MC3T3-E1 treated with zinc ions were observed. After culturing for 2 and 3 days, zinc ions had a positive effect on HUVECs, especially at 1 and 2 ppm. Over time, 1 and 2 ppm zinc ions significantly promoted the proliferation of MC3T3-E1 cells. However, when the concentration of zinc ions was up to 4 and 8 ppm, zinc ions promoted the proliferation of MC3T3-E1 in an unapparent manner, or even negatively. These results demonstrated that an appropriate dose of zinc ions was biologically safe for cells, whereas excessive zinc ions were cytotoxic, leading to apoptosis.

### Cell Adhesion and Spreading

**FIGURE 6:**
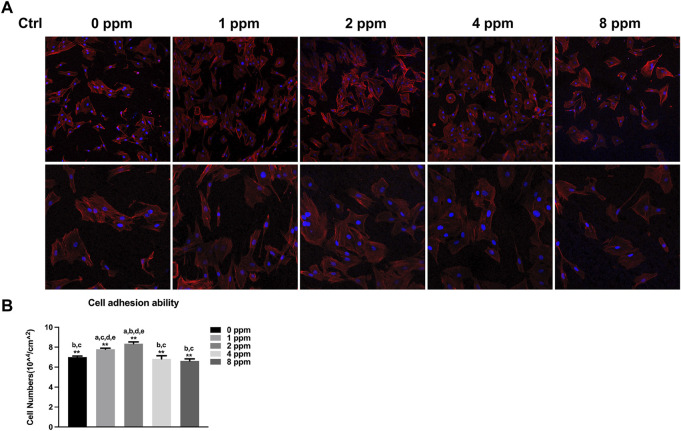
Cell adhesion ability assay. The adhesion ability of MC3T3-E1 cells was analyzed by counting the stained nuclei with DAPI using a confocal laser scanning microscope (CLSM) after incubation. **(A)** CLSM images of the cells under different zinc ions concentrations at ×100 and ×200 magnification. **(B)** Statistical results for adhesive cell numbers (*p < 0.05,**p < 0.01; N = 3).

**FIGURE 7:**
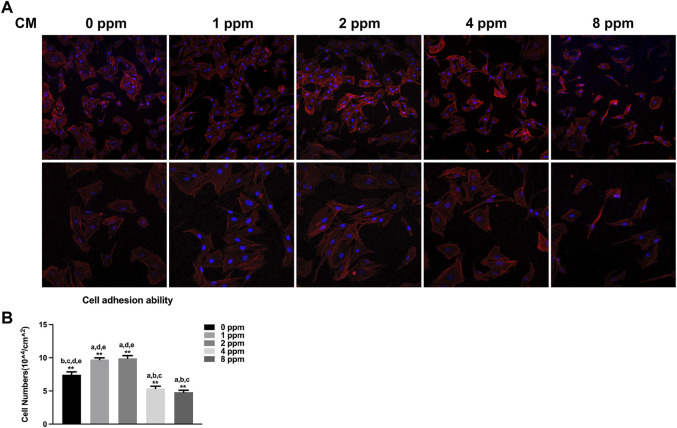
Cell adhesion ability assay. The adhesion ability of MC3T3-E1 cells was analyzed by counting the stained nuclei with DAPI using a confocal laser scanning microscope (CLSM) after incubation. **(A)** CLSM images of the cells under different concentrations of CM at ×100 and ×200 magnification. **(B)** Statistical results for adhesive cell numbers (*p < 0.05,**p < 0.01; N = 3).

When exposed to different doses of zinc ions, differences in the cell morphology could be observed under the laser scanning confocal fluorescence microscope (Figure 6). Compared with 0 ppm, more adherent cells under 1 and 2 ppm zinc ions seemed to be observed using a confocal laser scanning microscope (CLSM). There were no obvious differences in 1 and 2 ppm. After treating with CM, the number of adherent cells and the cell morphology were better than those of control groups without CM; in other words, CM could facilitate the cell adhesion significantly (Figure 7).

**FIGURE 8:**
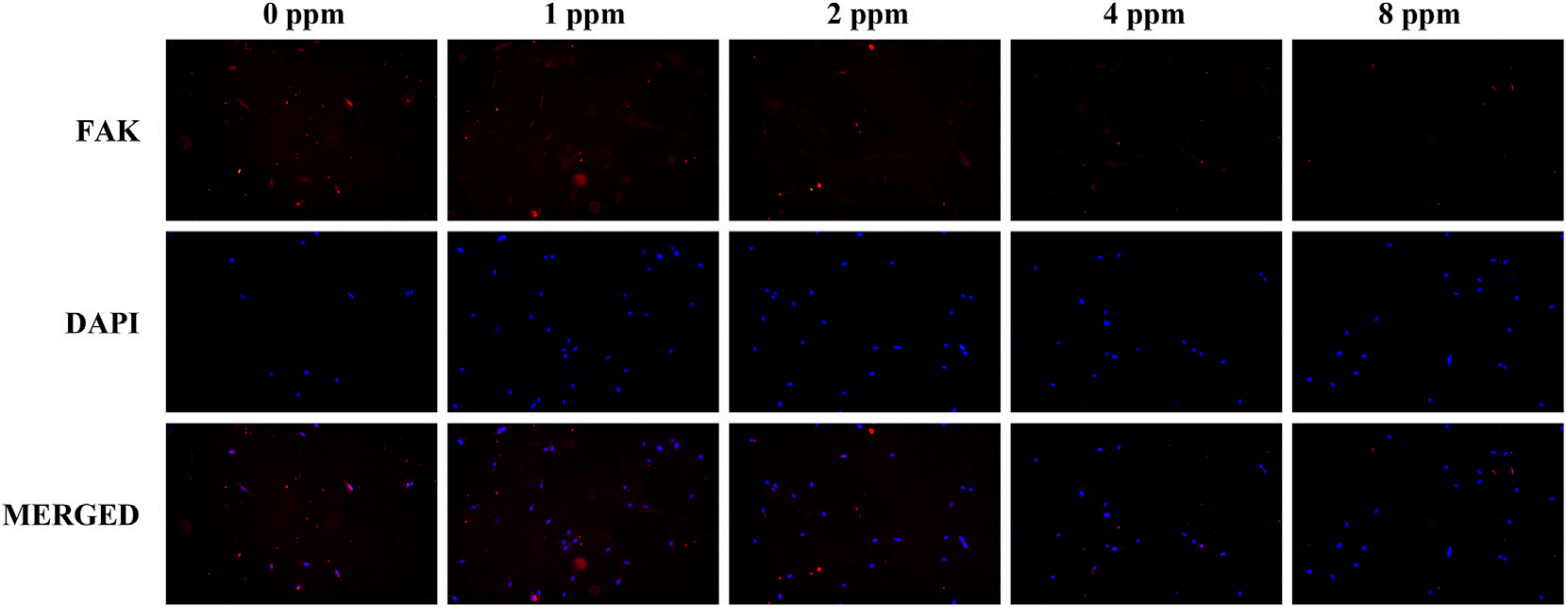
Immunolocalization of FAK (red) and nuclei (blue). The merged images show that the treatment of 1 and 2 ppm zinc ions promoted FAK expression significantly. All images are displayed at ×200 magnification.

**FIGURE 9:**
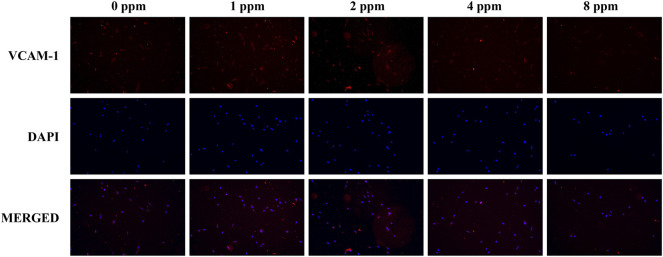
Immunolocalization of VCAM-1 (red) and nuclei (blue). The merged images show that the treatment of 1 and 2 ppm zinc ions promoted VCAM-1 expression significantly. All images are displayed at ×200 magnification.

Differences in the cell adhesion when exposed to different doses of zinc ions are presented in Figure 8 and Figure 9. Focal adhesion kinase (FAK) is a widely expressed cytoplasmic protein tyrosine kinase involved in cell adhesion and proliferation. Also, vascular cell adhesion molecule-1 (VCAM-1) plays a role in functioning as a cell adhesion molecule. As shown in Figure 8 and Figure 9, the fluorescence expression intensity of FAK and VCAM-1 in 1 and 2 ppm zinc ions was higher than that in other groups, which proved the positive effects under 1 and 2 ppm zinc ions in cell adhesion from another perspective.

### Western Blotting Analysis

**FIGURE 10:**
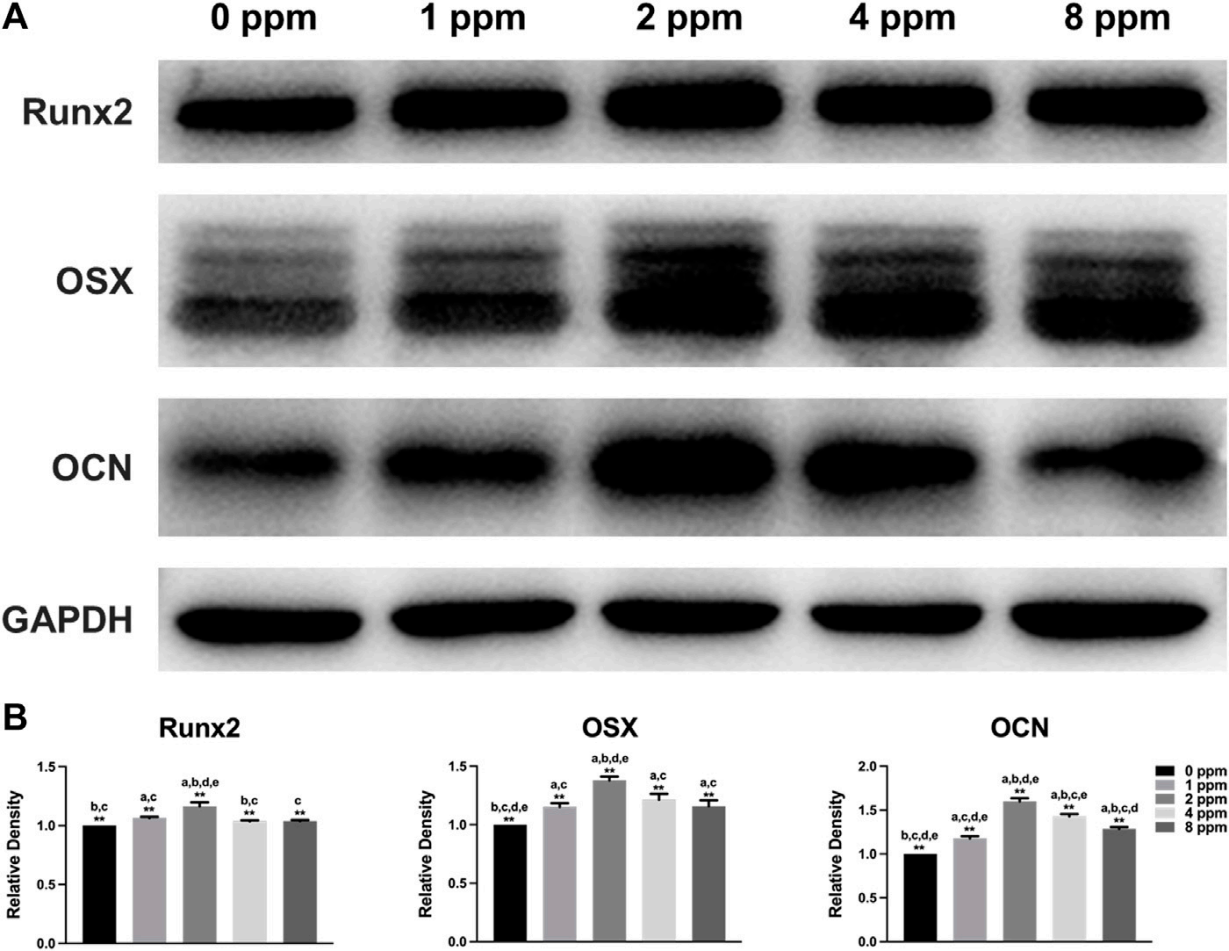
Protein expression levels of osteogenic markers Runx2, OSX, and OCN of osteoblast-like MC3T3-E1 cells in different concentrations of CM (Runx2, Runt-related transcription factor 2; OSX, osterix; OCN, osteocalcin). Quantification was performed by ImageJ software. Results are presented as mean ± SD (*p < 0.05, **p < 0.01; N = 3).

**FIGURE 11:**
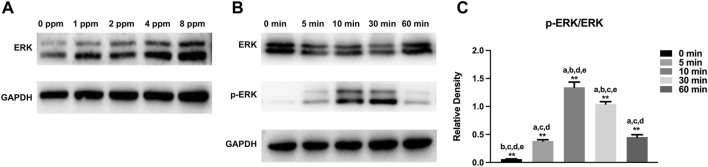
Activation of MAPK/ERK signaling by different concentrations of CM. **(A)** Protein expressions of vital members for the MAPK/ERK signaling pathway in MC3T3-E1 cells treated with different concentrations of CM. **(B)** Protein expressions of vital members for the MAPK/ERK pathway in MC3T3-E1 cells exposed to CM of 2 ppm at different time points. **(C)** Histogram showing normalized ratios of p-ERK/ERK. Results are presented as mean ± SD (*p < 0.05,**p < 0.01; N = 3).

Western blotting was performed to examine the osteogenic-related protein expression levels. Runx2 and OSX play a key role in the early stage of osteogenic differentiation and bone formation. In addition, OCN serves as a late marker of osteogenic differentiation and directly participates in the mineralization process. As shown in Figure 10, different doses of CM had different influences on cells. In this study, protein expression of Runx2, OSX, and OCN of experimental groups (1, 2, 4, and 8 ppm) was significantly higher than that of the control group (0 ppm). The conditioned medium, achieved from endothelial cells treated with 2 ppm zinc ions, showed the best osteogenic capacity. Hence, 2 ppm CM was chosen to culture osteoblasts to observe the expression of p-ERK. As one of the MAPK protein members, the expression of phosphorylated ERK protein peaked at 10 min. In Figure 11, as the concentration of the conditioned medium increased, the expression of ERK was found to be increased, indicating that the conditioned medium activated the MAPK/ERK signaling pathway.

## Discussion

Nowadays, surface modification of titanium implants has gained worldwide attention due to the development of oral implantology. Many methods of surface modification are proved to increase the roughness, wettability, and surface bioactive, thus enhancing osteoinduction and osteogenesis. Various methods have incorporated novel titanium surfaces with micro-, nano-, or micro-/nanostructures. Meanwhile, since zinc ions provide many advantages in biomedicine, there have been more and more studies on zinc-modified implants that have been primarily focused on their excellent ability to promote bone regeneration (Yusa et al., 2016). In our previous study, we successfully incorporated a novel acid-etched microstructured titanium surface modified with zinc-containing nanowires (Ti-NW-Zn), which proved that a zinc-modified titanium surface has good corrosion resistance and antibacterial activity. The excellent performance of zinc-modified implants was mostly due to the introduction of zinc ions (Shao et al., 2020).

Surface characterization is an important factor that helps in the improvement of implant properties, shortens healing times, and enhances osteointegration. As observed in SEM images, the Ti-NW-Zn surface featured porous nanostructures interweaved into networks. From XPS data, the Ti-NW-Zn surface mostly contained titanium, oxygen, carbon, and zinc. When inserted into the implant bed, the blood initially reaches the implant surface, which triggers subsequent biological behavior and suggested the significance of hydrophilicity. The hydrophilicity assay clearly revealed the modified titanium surfaces were superior to the control group, with contact angles of 55°, 45°, and 44°, respectively.

Zebrafish share 70 percent of their genetic homology with humans (Rothenbucher et al., 2019). Nowadays, the transgenic zebrafish model is commonly used to observe the early development of blood vessels (Lawson and Weinstein, 2002). Also, the physiological structure of the caudal fins on a zebrafish approximates that of a human dermal bone as both have strong regeneration abilities (Uemoto et al., 2020). Therefore, zebrafish models apply to the study of blood vessel development and bone regeneration *in vivo*. In this study, we established a Tg (Fli-1:EGFP)^y1^ zebrafish model to study the effects of zinc ions on angiogenesis and bone regeneration *in vivo* and further analyzed the effects of vascular endothelial cells on osteoblasts in the presence of zinc ions through *in vitro* cell models to reveal the specific mechanism between angiogenesis and osteogenesis upon exposure to zinc ions.

Our previous study proved that modified titanium surfaces accelerated dental implant osseointegration and showed good biocompatibility. In this study, Ti-NW-Zn promoted both angiogenesis, when pretreated with VRI in the Tg (Fli-1:EGFP)^y1^ zebrafish model (Figure 2), and osteogenesis in the zebrafish fin amputation model (Figure 3A), which demonstrated that zinc ions played a vital role in angiogenesis and osteogenensis. We also found these positive effects depended on the zinc ion concentration; 1 ppm of zinc better promoted fin regeneration than the control group and 2 ppm zinc (Figure 3B). In order to more accurately identify the number of zinc ions released from the Ti-NW-Zn surface, a zinc assay kit was used, which determined the concentration of zinc ions released from the Ti-NW-Zn surface was ∼1 ppm (Figure 1E), which agreed with the appropriate zinc ion concentration range that could promote zebrafish fin regeneration. We assessed the survival curve of embryos and adult fish under zinc ion exposure to evaluate the biological safety of zinc ions and zebrafish and determine the optimized concentration range of zinc ions for zebrafish angiogenesis and bone regeneration (Figure 4). After exposure to zinc, the median lethal concentration (LC) in 24 hpf-zebrafish embryos was >30 and ∼2 ppm for adult fish. Although the survival rates in different periods differed significantly, zebrafish embryos chronically exposed to zinc ions developed a higher tolerance than adult fish, and the concentration of zinc ions released from the Ti-NW-Zn surface detected above was much lower than the median lethal concentrations (LCs) of both embryos and adult fish. These results suggested that zinc ion concentration released from the Ti-NW-Zn surface was appropriate and safe to promote angiogenesis, osteogenesis, and the survival rate of zebrafish. Furthermore, the zebrafish model was successfully applied in the study of blood vessel development and bone regeneration exposure to zinc ions.

Although studies have shown that zinc ions not only affect the mineralization of osteoblasts by participating in the formation of bone salt (Nagata and Lonnerdal, 2011) but also promote migration of vascular endothelial cells and maintained proliferation; the growth and active states of apoptotic balance promoted skeletal remodeling (Pan et al., 2020; Xia et al., 2020) and suggested that a significant pathway for zinc ion promotion of bone regeneration may stem from blood vessel development in the bone. Other questions to consider is how do zinc ions affect angiogenesis and mineralization *in vitro*? What is the relationship between angiogenesis and osteogenesis under zinc ions exposure? In our *in vitro* cell models, we explored the effects of vascular endothelial cells on osteoblasts upon exposure to zinc ions. As shown in Figure 5, zinc ion concentrations of 1 and 2 ppm both had positive effects on the proliferation of endothelial cells and osteoblast compared with the control group. Cell adhesion and spreading are vitally important in regulating cell behaviors (Gumbiner, 1996). Figure 6 shows the results from our exploration into the relationship between zinc ions and the adhesion morphology of osteoblast; zinc ion concentrations between 1 and 2 ppm better promoted cell spreading and pseudopodia extensions. Interestingly, the number and morphology of MC3T3 treated with conditioned medium (CM) surpassed the those of the control group without CM (Figure 7), which indicated that CM improved cell adhesion significantly. More and more studies have reported that some cytokines and growth factors that endothelial cells secrete had impacts on osteoblasts, such as the most important pro-angiogenic factor, vascular endothelial growth factor (VEGF), and the key regulatory factor of VEGF, hypoxia-inducing factor (HIF) (Schipani et al., 2009; Diomede et al., 2020). These results and reports suggested that appropriate levels of zinc promote osteoblast behavior by inducing the secretion of beneficial substances from endothelial cells. We also found the promotion of cell differentiation under the exposure of 2 ppm CM as the expressions of osteogenic markers were all upregulated (Figure 10). These data suggested that lower concentrations of zinc positively regulated osteoblast behaviors, while higher concentrations had negative effects.

MAPK signaling pathways have been recognized as important regulators of bone mass and osteoblast differentiation (Wang et al., 2014). Different stresses activate MAPK and influence apoptosis either positively or negatively (Yue and Lopez, 2020). In many cells, ERK inhibits apoptotic processes, whereas JNK and p38 signaling pathways contribute to the induction of apoptosis (Sui et al., 2014; Guo et al., 2016; Kim et al., 2019). In Figure 11, the expression of ERK increased with increasing Zn ion concentrations. We found that 2 ppm CM optimized osteogenesis. Hence, CM containing 2 ppm Zn was selected for subsequent experiments to explore the role of the MAPK/ERK signaling pathway in osteoblast behaviors and notice the expression of p-ERK under exposure to Zn ions. Interestingly, p-ERK is rapidly phosphorylated, and activation peaked after 10 min before decreasing. Generally speaking, MAPK maintained normal levels (Guo et al., 2017). Zinc ions, acting as an extracellular, stimulated the upstream of the MAPK signaling axis, activated the phosphorylation of MAPK, and facilitated the downstream cascade reaction. Phosphorylation was transient within 60 min and rapidly returned to normal. Once activated, the MAPK signaling pathway responded and induced further reactions in a cascade fashion and ultimately affected the regulation of osteogenic markers (Sun et al., 2015; Zhu et al., 2019b). These results indicated the MAPK/ERK signaling pathway might participate in the osteoblast behavior upon Zn exposure though the specific mechanism requires additional study.

## Conclusion

In summary, we developed an innovative zinc-modified titanium surface which showed a good angiogenic and osteogenic capacity. Zinc ions played a key role in this improved titanium surface. The results revealed that the appropriate concentration of zinc ions could promote angiogenesis and osteogenesis *in vivo* and *in vitro* and could activate the MAPK/ERK signaling pathway, which affects osteoblast differentiation. These findings provided a theoretical basis to further improve the new titanium surface with appropriate zinc ion concentration.

## Data Availability

The raw data supporting the conclusion of this article will be made available by the authors, without undue reservation.

## Abbreviations

CM, conditioned medium; cp-Ti, commercially pure titanium; Ti-NW-Zn, titanium surface modified with zinc-containing nanowires.
