# Supplementary figures and images for: Effects of Zinc Ions Released From Ti-NW-Zn Surface on Osteogenesis and Angiogenesis In Vitro and in an In Vivo Zebrafish Model
Source: Front Bioeng Biotechnol. 2022 Apr 21;10:848769. doi: 10.3389/fbioe.2022.848769 (PMC9068938; doi:10.3389/fbioe.2022.848769)

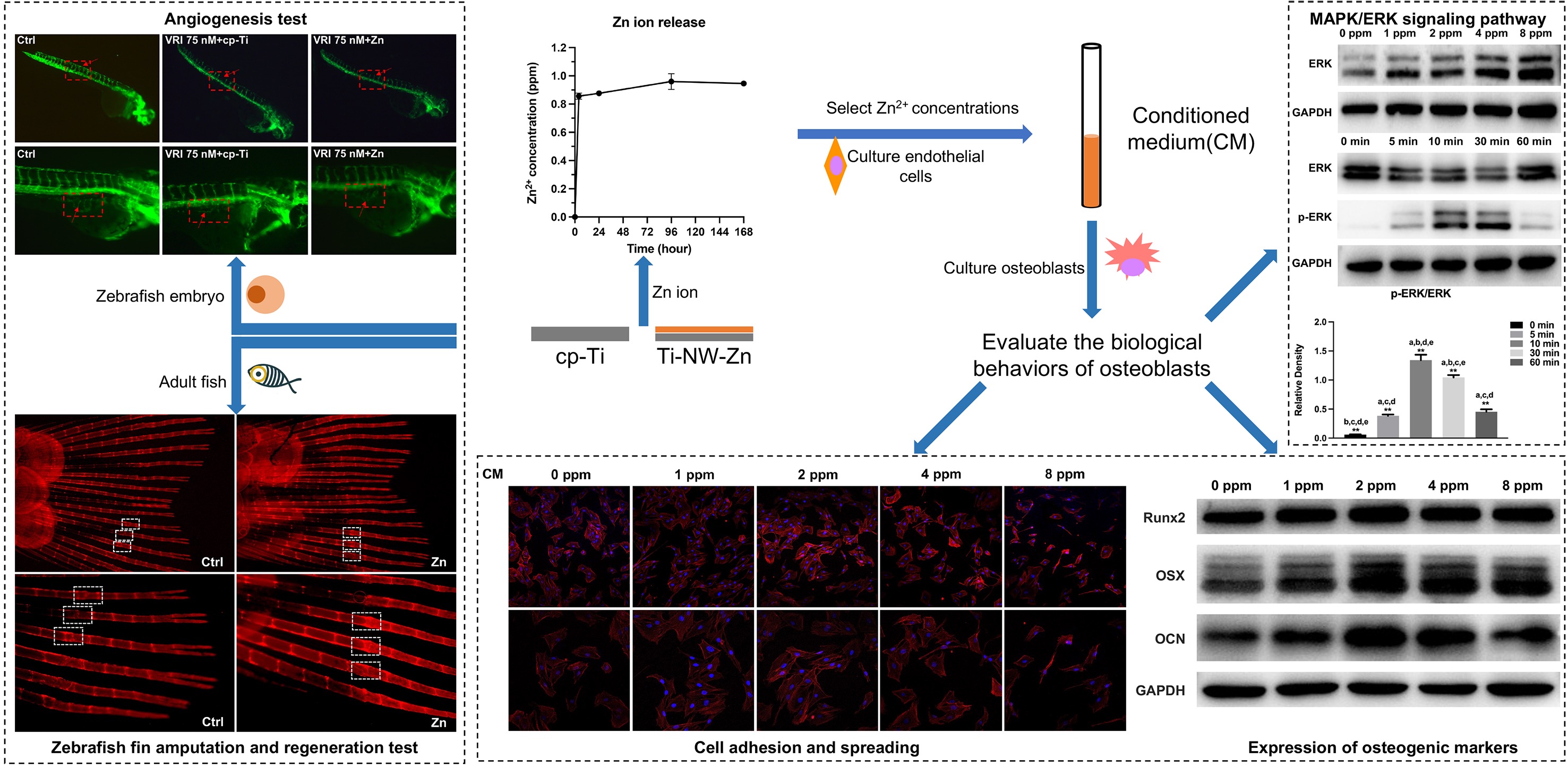

Supplement: Supplementary file 1 [file Image1.TIF]
